# Supplementary figures and images for: A highly contiguous genome assembly of red perilla (Perilla frutescens) domesticated in Japan
Source: DNA Res. 2022 Nov 16;30(1):dsac044. doi: 10.1093/dnares/dsac044 (PMC9835750; doi:10.1093/dnares/dsac044)

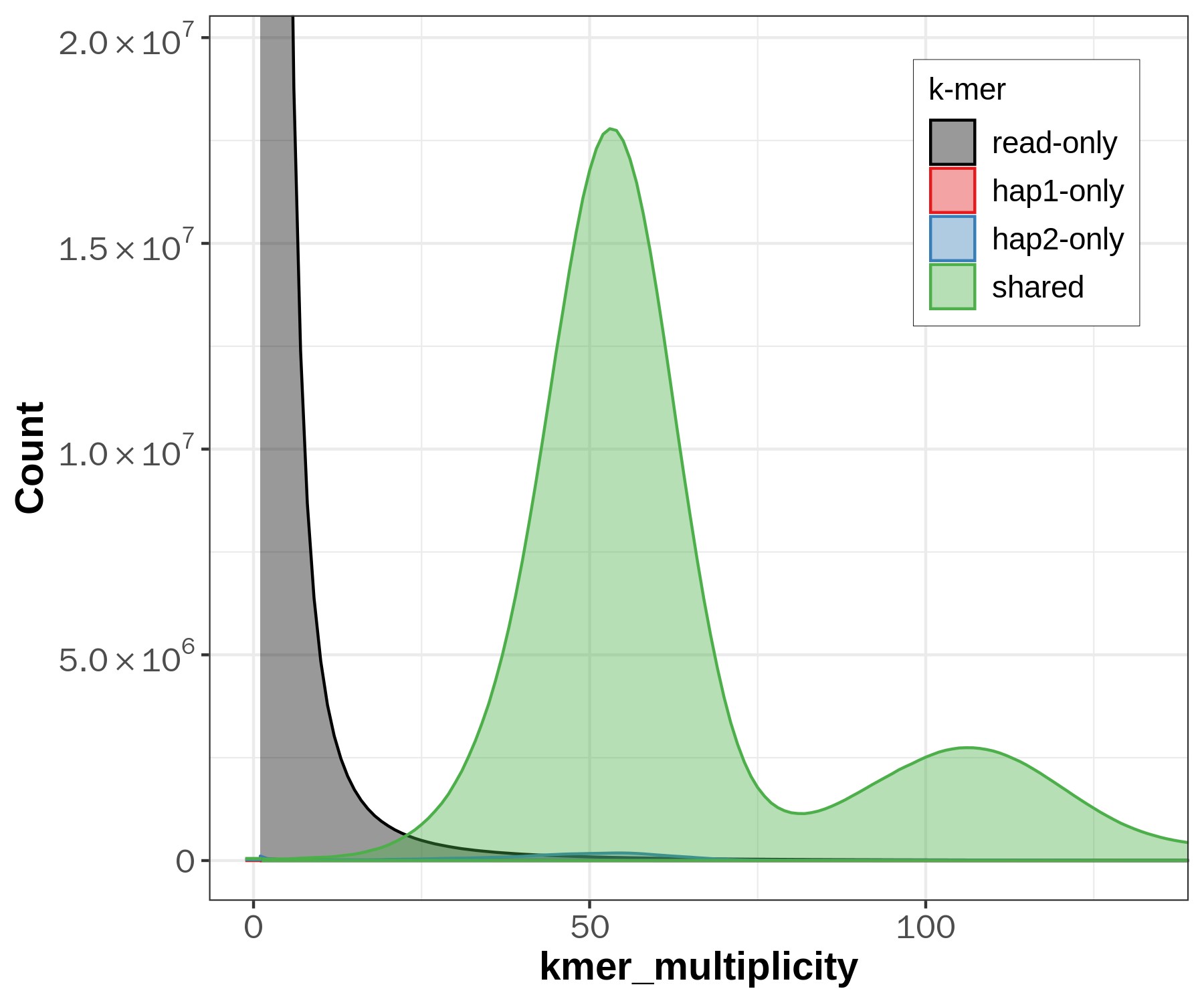

Supplement: dsac044_suppl_Supplementary_Figure_S1 [file dsac044_suppl_supplementary_figure_s1.jpeg]

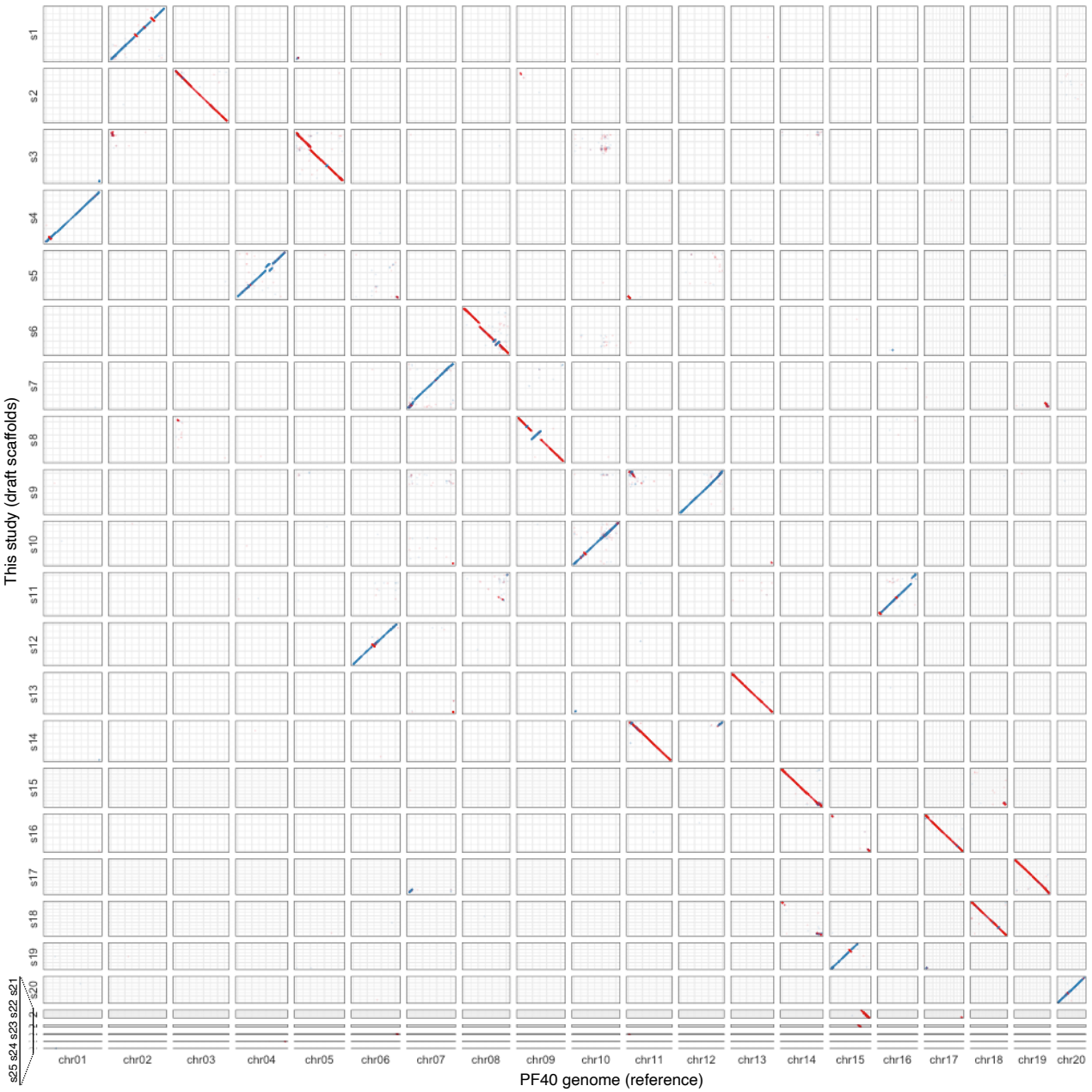

Supplement: dsac044_suppl_Supplementary_Figure_S2 [file dsac044_suppl_supplementary_figure_s2.pdf]

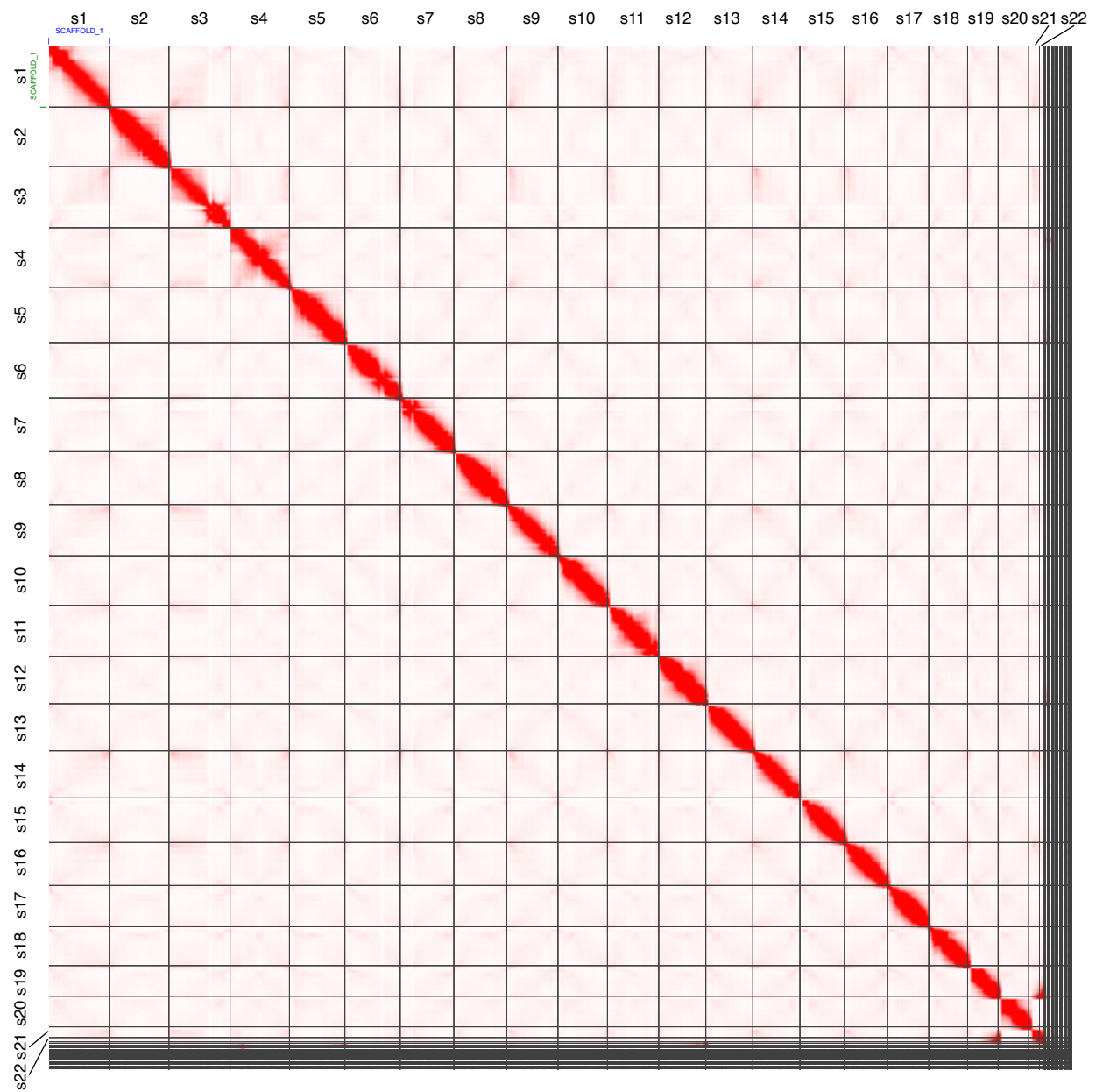

Supplement: dsac044_suppl_Supplementary_Figure_S3 [file dsac044_suppl_supplementary_figure_s3.pdf]
