# Supplementary material for: A highly contiguous genome assembly of red perilla (Perilla frutescens) domesticated in Japan
Source: DNA Res. 2022 Nov 16;30(1):dsac044. doi: 10.1093/dnares/dsac044 (PMC9835750; doi:10.1093/dnares/dsac044)
Supplement: dsac044_suppl_Supplementary_Table_S1 [file dsac044_suppl_supplementary_table_s1.docx]

**Supplementary Table S1.** Source of protein-level functional annotation of Pfru_yukari_1.0

| Annotation source | Source URL |
| --- | --- |
| Arabidopsis | http://ftp.ensemblgenomes.org/pub/release-53/plants/fasta/arabidopsis_thaliana/pep/Arabidopsis_thaliana.TAIR10.pep.all.fa.gz |
| Rice | http://ftp.ensemblgenomes.org/pub/release-53/plants/fasta/oryza_sativa/pep/Oryza_sativa.IRGSP-1.0.pep.all.fa.gz |
| Tomato | http://ftp.ensemblgenomes.org/pub/release-53/plants/fasta/solanum_lycopersicum/pep/Solanum_lycopersicum.SL3.0.pep.all.fa.gz |
| Human | https://ftp.ensembl.org/pub/release-105/fasta/homo_sapiens/pep/Homo_sapiens.GRCh38.pep.all.fa.gz |
| Mouse | https://ftp.ensembl.org/pub/release-105/fasta/mus_musculus/pep/Mus_musculus.GRCm39.pep.all.fa.gz |
